# The rise of RAS: how gradual oncogene activation shapes the OIS spectrum

**DOI:** 10.1101/gad.352761.125

**Published:** 2025-08-01

**Authors:** Haoran Zhu, Adelyne Sue Li Chan, Masashi Narita

**Affiliations:** Cancer Research UK Cambridge Institute, Li Ka Shing Centre, University of Cambridge, Cambridge CB2 0RE, United Kingdom

**Keywords:** oncogene-induced senescence, RAS, tumor-initiating cells, liver cancer

## Abstract

Excessive levels of oncogenic RAS expression in normal cells trigger reactive cellular senescence, known as oncogene-induced senescence (OIS)—a putative autonomous tumor-suppressive mechanism. However, the monoallelic expression of oncogenic RAS from the endogenous locus often fails to induce senescence, at least in the short term. Consequently, whether robust senescence characterizes the preneoplasia driven by oncogenic RAS under physiological conditions has been debated. A key challenge is the highly heterogeneous nature of senescence at both the population and single-cell levels. Notably, increasing evidence suggests that RAS levels are gradually upregulated during the development of tumors driven by oncogenic RAS. To address the complex relationship between diverse oncogenic responses, including senescence and tumor initiation, we introduce the concept of an OIS spectrum, where oncogenic dosage-dependent cellular states lie between normal cells and full senescence. Intermediate “sub-OIS” states may play a critical role in tumor initiation, potentially providing one explanation for the ongoing debate.

## Abstract (precis)

In this Perspective, Zhu et al. discuss the complexity and duality of oncogene-induced senescence (OIS), addressing an existing debate in the field about the role of OIS in normal physiology and tumorigenesis. The authors posit that oncogenic RAS, in a dosage-dependent manner, drives intermediate cellular states of OIS (termed the “OIS spectrum”), which play a critical role in tumor initiation.

Cellular senescence is a collective phenotype representing a state of highly stable cell cycle arrest. It was first described as replicative senescence in the early studies of Hayflick and Moorhead (1961), who observed that human diploid fibroblasts (HDFs) in culture undergo a limited number of divisions before entering an “irreversible” arrest state. Replicative senescence is closely linked to telomere attrition, where critically short telomeres are recognized as double-strand DNA damage (Shay and Wright 2000). Although initially described as a consequence of telomere shortening, senescence is now recognized as a cellular response to a broad range of stressors, though senescence-like phenotypes have also been observed in physiological contexts, such as embryonic development (Chuprin et al. 2013; Muñoz-Espín et al. 2013; Storer et al. 2013). Senescence triggers include DNA damage, oxidative and metabolic stress, excessive oncogenic activation, and other cytotoxic agents (Salama et al. 2014; Zhu et al. 2020). Among these, oncogene-induced senescence (OIS) stands out as a paradoxical putative tumor-suppressive mechanism (see below).

Senescence also involves a diverse set of effector programs, with different programs activated depending on the context (e.g., the trigger and the cell type), contributing to the induction, amplification, and maintenance of senescence. These different effector programs often reflect individual senescence markers and contribute to the heterogeneity of the senescence phenotype (Salama et al. 2014). These markers include, but are not limited to, upregulation of endogenous cyclin-dependent kinase inhibitors (e.g., p16 and p21), persistent activation of DDR (e.g., γH2AX and 53BP1 foci), enhanced lysosomal activities (senescence-associated β-galactosidase activity), altered secretory profiles (e.g., secretion of proinflammatory factors) known as the senescence-associated secretory phenotype (SASP), mitochondrial dysfunction, and nuclear and chromatin alterations (e.g., senescence-associated heterochromatic foci [SAHFs], senescence-associated distension of satellites [SADS], and cytoplasmic chromatin fragments [CCFs]) (Swanson et al. 2015; Miller et al. 2021; Mylonas and O'Loghlen 2022). Of note, these markers are not definitive by themselves. For example, SAHF formation is more prominent in OIS, whereas SASP is less pronounced in p16-induced senescence in HDFs (Narita et al. 2003; Zhang et al. 2005; Rodier et al. 2009). Therefore, senescence is typically identified through a combination of these context-dependent markers (Gorgoulis et al. 2019; Suryadevara et al. 2024).

We recently proposed a complemental view of senescence as a stress-responsive fate determination process mediated through an altered high-order epigenetic landscape (akin to terminal differentiation) (Olan and Narita 2022; Olan et al. 2023). Cellular functionality is intricately linked to cell fate specification and lineage determination. Although intracellular proteins contribute to unique cellular functions and cell type specificity, tissue-specific genes are best represented in cell surface and secretory factors (Olan and Narita 2022; Olan et al. 2023). A hallmark of senescence, beyond stable proliferative arrest, is its significant non-cell-autonomous activities, playing a central role in intercellular communication and modulation of the surrounding environment through various mechanisms in an either direct (juxtacrine) or indirect (autocrine and paracrine) manner (Fafián-Labora and O'Loghlen 2020; Olan and Narita 2022). The SASP in particular involves extensive alterations in soluble as well as extracellular vesicle secretory profiles (Birch and Gil 2020; Fafián-Labora and O'Loghlen 2020; Wallis et al. 2020; Takasugi et al. 2023).

Although the SASP usually refers to the upregulation of certain factors, including upregulation of inflammatory cytokines, chemokines, extracellular matrix (ECM) remodelers, and growth factors, it can also involve the repression of secretory factors characteristic of specific cell types. For example, senescence in fibroblasts is typically accompanied by reduced production of type I collagen and other ECM components, suggesting a reduction of the inherent fibrogenic activity (Levi et al. 2020). A similar shift in functional identity has been noted in aging contexts, where dermal fibroblasts from older mice show reduced expression of similar ECM-forming genes but instead gain adipogenic characteristics (Salzer et al. 2018). Together, these support the notion that senescence can be seen as a state of altered functionality, substantially deviating from the cell of origin. What is the mechanism behind this?

The acquisition of a new functional identity occurs during cell differentiation through refined alterations in gene expression, particularly involving tissue-specific genes that are tightly regulated by epigenomic mechanisms. Similarly, senescence is associated with extensive alterations in high-order chromatin architecture at various levels (Olan and Narita 2022; Olan et al. 2023). Using the HDF OIS model (see below), we have recently shown that senescent cells exhibit a striking rewiring of the enhancer–promoter networks (Olan et al. 2020), which can control tissue-specific gene regulation (Javierre et al. 2016; Rubin et al. 2017). Interleukin-1α (IL1α) and ILβ, major SASP factors, are primarily produced by immune cells like macrophages, but the *IL1* locus (containing *IL1A* and *IL1B*) exhibits new chromatin loop formation in OIS HDFs, promoting specific enhancer–promoter binding (Olan et al. 2020). Interestingly, the new loop structure resembles that of terminally differentiated macrophages of THP-1 monocytes (Olan and Narita 2022). Importantly, when HDFs are acutely stimulated with TNFα, which triggers stabilization and nuclear translocation of the essential transcription factor NF-kB to induce *IL1A* and *ILB* expression, such 3D chromatin changes do not occur (Jin et al. 2013), suggesting that *IL1A* and *IL1B* regulation as SASP components is fundamentally different from the mechanism that occurs acutely in HDFs.

We recently extended this idea to more strict lineage-dependent genes that are highly heterochromatic in HDFs. The epidermal differentiation complex (EDC) is an ∼2 Mb gene cluster in human chromosome 1q21, and EDC genes encode structural and functional components of terminal differentiation; i.e., cornification of keratinocytes (Qin et al. 2022). The EDC locus is tightly condensed with a heterochromatic marker, histone H3K9me3, in normal HDFs. However, in OIS HDFs, it becomes physically decondensed, and chromatin loops that are more similar to terminally differentiated keratinocytes appear. Some EDC genes are indeed derepressed in OIS HDFs (Tomimatsu et al. 2022).

These studies suggest that fully established senescence is associated with chromatin architectures characteristic of terminally differentiated states (extensively discussed in our recent reviews) (Olan and Narita 2022; Olan et al. 2023). This raises a key question: What is the “trajectory” of this process and how does it relate to associated plasticity? In this Perspective, we focus on OIS, which not only is dynamic but also exhibits substantial heterogeneity at steady state. We first provide an overview of the oncogenic RAS response in normal cells, emphasizing its dual role in either tumor suppression (i.e., OIS) or tumor initiation, the complexity largely driven by oncogenic dosage. To address this issue, we introduce the concept of the OIS spectrum, which encompasses shifts in cellular plasticity and may help reconcile these opposing outcomes.

## Oncogene-induced senescence

**Figure 1.**
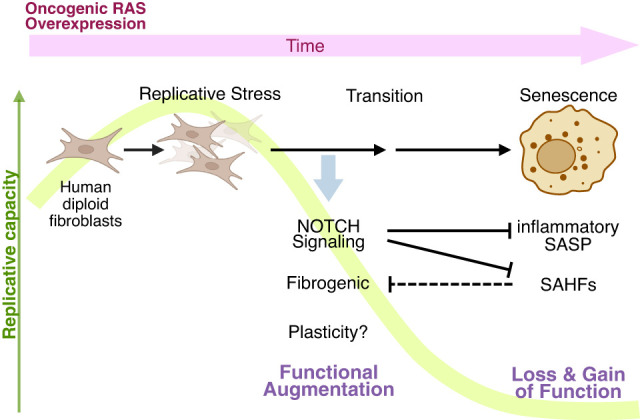
The classic OIS model in culture exhibits a dynamic and progressive phenotype. Induction of ectopic oncogenic RAS drives hyperproliferation, leading to replication-associated DNA damage. This is followed by a transition phase characterized by transient activation of NOTCH and TGFβ signaling, correlating with increased fibrogenic features (“functional augmentation” in fibroblasts). NOTCH signaling negatively regulates the inflammatory component of the SASP and SAHF formation, contributing to senescence heterogeneity. In the established phase, SAHFs encompass various genes associated with fibrogenic features, which become downregulated (“loss of function” in OIS cells), whereas the inflammatory SASP exemplifies a gain of function. How NOTCH signaling influences cellular plasticity in this context remains unclear. (OIS) Oncogene-induced senescence, (SASP) senescence-associated secretory phenotype, (SAHFs) senescence-associated heterochromatic foci.

Oncogene-induced senescence (OIS) is a paradoxical response triggered by the aberrant activation of certain oncogenes. As originally identified in HDFs in culture, OIS is best exemplified by ectopic expression of a constitutively active form of RAS or downstream effectors in the MAPK pathway (Fig. 1; Serrano et al. 1997; Lin et al. 1998; Zhu et al. 1998). The RAS family of small GTPases plays a central role in transducing extracellular signals to regulate cell growth, differentiation, and survival. Under physiological conditions, RAS cycles between an inactive GDP-bound state and an active GTP-bound state in response to mitogenic signals. However, oncogenic mutations, such as the common G12V or G12D substitutions in RAS, lock RAS in a constitutively active state.

OIS is a highly dynamic and progressive process: Ectopic expression of oncogenic RAS in HDFs in culture initially stimulates cell division, which is followed by a gradual loss of proliferative capacity and the accumulation of senescence features (Young et al. 2009). The process—the initial excessive cell proliferation—leads to replication fork stalling and a DNA damage response, thus contributing to the senescence phenotype (Bartkova et al. 2006; Micco et al. 2006; Mallette et al. 2007). Oncogenic RAS expression has also been suggested to increase reactive oxygen species, contributing to senescence induction (Lee et al. 1999).

The dynamic process of OIS is in part controlled by NOTCH signaling (Ito et al. 2017). We have shown that NOTCH signaling is activated during OIS, particularly around the transition between proliferative and stably arrested phases. The “NOTCH phase” in HDF OIS exhibits upregulation of TGFβ signaling and other fibrogenic features that, as mentioned above, are typically downregulated in the established senescence phase. Thus, fibroblasts exhibit a transient “functional augmentation” during OIS. In addition, NOTCH signaling suppresses inflammatory components of the SASP in part by inhibiting expression of the key transcription factor C/EBPβ, contributing to mutually exclusive profiles between fibrogenic and inflammatory secretory features (Hoare et al. 2016). Therefore, NOTCH signaling regulates the dynamic and timely shift of cell functionality during senescence (Fig. 1). It remains to be elucidated whether such functional augmentation is also controlled through high-order chromatin architecture. NOTCH activation has also been reported in different senescence contexts, suggesting that NOTCH-mediated modulation of senescence might be relatively common (Hoare and Narita 2018).

Non-cell-autonomous activities of senescence also control the spatial heterogeneity of the senescence phenotype. For example, SASP factors can promote senescence in adjacent cells via amplifying inflammatory signaling (Acosta et al. 2008, 2013; Kuilman et al. 2008). This effect is not limited to soluble factors, as extracellular vesicles from senescent cells can also contribute to the spatial spreading of the signals with the cargos, including proteins (e.g., IFITM3) and noncoding RNAs (ncRNAs; e.g., pericentromeric ncRNA) (Borghesan et al. 2019; Miyata et al. 2021; Oh et al. 2022). In addition, NOTCH signaling is mediated by direct cell–cell contact, wherein cell surface ligands (e.g., JAG1) bind cell surface NOTCH receptors (Hoare et al. 2016; Parry et al. 2018; Teo et al. 2019). Upon ligand–receptor binding, NOTCH receptors undergo serial proteolytic cleavage, translocate into nuclei, and control NOTCH-responsive gene expression. Interestingly, NOTCH activation upregulates the ligand JAG1 in the context of HDF senescence, thus reinforcing NOTCH signaling in adjacent cells (“lateral induction”) (Hoare et al. 2016). Moreover, NOTCH signaling also inhibits SAHF formation partly by suppressing the expression of HMGA1, an essential structural component of SAHFs (Narita et al. 2006; Parry et al. 2018). Therefore, the senescence phenotype, including the SASP composite and SAHFs, is highly heterogeneous, and NOTCH signaling contributes to it in a temporospatial manner.

## OIS in physiological conditions

The OIS observed in cultured HDFs is generally achieved through the forced expression of ectopic oncogenic RAS. Therefore, the level of oncogenic RAS expression is considerably higher compared with endogenous RAS, leaving the question of whether OIS occurs under more physiological conditions. Mutations in oncogenes such as RAS typically begin with a mutation in a single allele in spontaneous cancers. Indeed, it has been shown that a single copy of oncogenic RAS, regulated by an endogenous promoter, fails to induce senescence (Guerra et al. 2003; Hingorani et al. 2003).

In the early 2000s, two mouse models harboring oncogenic *Kras* (either *Kras*^*K12D*^ or *Kras*^*G12V*^) under endogenous control in a Cre recombinase-dependent manner were independently generated (Jackson et al. 2001; Guerra et al. 2003). Mouse embryonic fibroblasts (MEFs) are generally sensitive to oxidative stress, and under atmospheric culture conditions, they rapidly exhibit changes resembling cellular senescence. However, MEFs derived from these mice did not show these changes and were in a state of immortality (Guerra et al. 2003; Tuveson et al. 2004). Therefore, endogenous oncogenic Kras does not induce cellular senescence, at least in the cultured MEF system. MEFs expressing endogenous Kras^G12V^ still undergo senescence in response to the additional dosage of oncogenic RAS, suggesting that the dosage of oncogenic stimuli may be critical to senescence induction. Consistently, low levels of ectopic Raf, a downstream kinase of RAS, promote cell cycle progression, whereas higher Raf activity induces cell cycle arrest in MEFs (Woods et al. 1997).

Although the lack of senescence in MEFs is common in both models, there are some differences in phenotype in vivo. Tuveson et al. (2004) showed that widespread expression of endogenous *Kras*^*G12D*^ results in early embryonic lethality. However, Guerra et al. (2003) reported that a substantial number of mice with ubiquitous expression of endogenous *Kras*^*G12V*^ reach adulthood but go on to develop a broad spectrum of lung tumors. Subsequently, Collado et al. (2005) detected senescence features in lung adenomas, but not in adenocarcinomas, in this model. They also showed an increase in senescence markers in pancreatic intraductal neoplasias (PanINs) when endogenous *Kras*^*G12V*^ was specifically expressed in the pancreas. In contrast, Tuveson and colleagues (Tuveson et al. 2004; Caldwell et al. 2012) failed to detect senescence markers such as p53, p21, p16, and p19arf in the preneoplastic lesions of the pancreas, lung, or colon in the endogenous *Kras*^*G12D*^ model, though a subsequent more extensive analysis on OIS identified ∼10% of PanIN cells as positive for senescence-associated β-galactosidase (SA-β-gal) activity. Kolodkin-Gal et al. (2022) also reported similar results using the same mouse model.

Various technical factors might contribute to the apparent difference between the models, such as the use of different gene targeting strategies. For example, these mutations (*Kras*^*G12D*^ or *Kras*^*G12V*^) may have different activities (Li et al. 2018), and the *Kras*^*G12V*^ knock-in model also harbors the IRES-β-geo cassette at the 3′ untranslated region of endogenous *Kras*^*G12V*^, which might affect the stability of the gene (Guerra et al. 2003). However, it is likely that the intersample and intrasample heterogeneity of the phenotype can also be an explanation. Consistently, a recent single-cell transcriptome analysis of a pancreatic tumor model driven by endogenous *Kras*^*G12D*^ identified a small cluster with an OIS signature in preneoplastic lesions (Schlesinger et al. 2020). They used the same *Kras*^*G12D*^ mice, but *Kras*^*G12D*^ was somatically activated using tamoxifen-inducible Cre recombinase in the pancreas. In addition, recent studies highlighted the importance of senescent stromal cells in promoting tumorigenesis in oncogenic Kras-driven lung tumor models with both *Kras*^*G12D*^ and *Kras*^*G12V*^ mutations (Haston et al. 2023; Prieto et al. 2023). They detected senescent cells in preneoplastic lesions using p16 reporter systems, but the majority of p16-positive cells were stromal cells (macrophages in particular), and p16-positive epithelial cells were rare.

Altogether, endogenous oncogenic Ras promotes cell proliferation and does not induce senescence in vitro. However, at the organism level, some tissues develop preneoplastic lesions, which are heterogeneous and can include a small fraction of classical OIS cells.

## Oncogenic RAS dose variation during tumorigenesis

**Figure 2.**
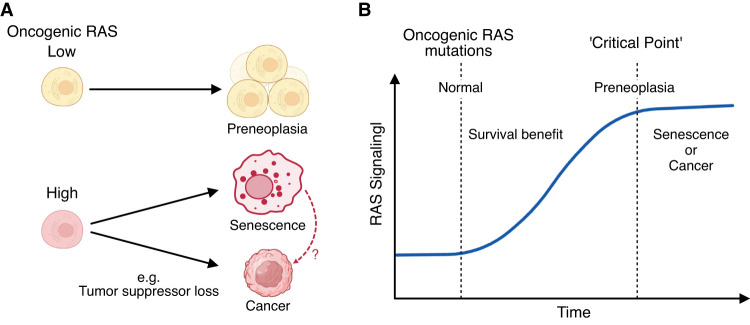
Oncogenic RAS dose-dependent phenotype shapes the OIS spectrum. (*A*) Generally, low levels of ectopic expression of oncogenic RAS promote cell proliferation, whereas excessive levels can induce senescence or promote tumorigenesis if senescence is bypassed. In this model, “full” senescence is tumor-suppressive, making direct malignant transformation less likely (dashed arrow). (*B*) Spontaneous upregulation of oncogenic RAS levels/activity. Endogenous levels of oncogenic RAS can be gradually upregulated due to its proliferative and survival benefit until it hits the “critical point,” which either triggers senescence or increases the risk for malignant transformation depending on the other factors. The mechanism of RAS upregulation remains unclear but often correlates with copy number duplication. Other factors, such as epigenetic regulation and signaling feedback, may also play a role.

Endogenous levels of oncogenic Kras do not immediately induce tumor formation in mice, and the frequency of malignancy is relatively low (Guerra et al. 2003; Hingorani et al. 2003). Thus, although endogenous oncogenic Ras may not be autonomously sufficient to induce a robust senescence phenotype in vivo, the same argument may also apply to cancer development. Indeed, the requirement for high oncogenic RAS expression in both senescence induction and cancer development has been demonstrated in an *Hras*^*G12V*^ transgenic mouse breast cancer model (Sarkisian et al. 2007). In this study, low levels of Ras activation triggered cellular proliferation and hyperplasias but not senescence. In contrast, high levels of Ras activation—similar to levels found in tumors with spontaneous (endogenous) *Kras* mutations in a Myc-driven mouse mammary tumor model—induced Ink4a/Arf-dependent senescence. The data suggest that a high level of Ras activity is required for both senescence induction and tumor initiation, but the latter occurs particularly when additional events, such as the loss of tumor suppressor genes, take place (Fig. 2A; Sarkisian et al. 2007).

Consistent with this idea, spontaneous upregulation of RAS–MAPK signaling has been observed in various tumor types in mice and humans (Burgess et al. 2017). For example, in endogenous *Kras*^*G12D*^-driven lung tumor models, MAPK signaling downstream from Ras is markedly higher in adenocarcinomas compared with adenomas (Feldser et al. 2010; Junttila et al. 2010). This is often accompanied by a copy number duplication of *Kras*^*G12D*^ with or without loss of wild-type alleles (Junttila et al. 2010). A similar allelic imbalance increase in oncogenic *Ras* dosage through copy number gains has been associated with acute myeloid leukemia (AML) in mice (Xu et al. 2013; Burgess et al. 2017) and pancreatic tumor development in mice and humans (Mueller et al. 2018; Varghese et al. 2025). Furthermore, in the systematic analysis of human cancer cell lines and tissues, levels of *RAS* transcripts are generally higher when *RAS* mutation is present (Xu et al. 2013; Stephens et al. 2017). Burgess et al. (2017) have identified 55% of KRAS mutant advanced cancers exhibiting allelic imbalance at the *KRAS* locus across 30 cancer types. We also reanalyzed the aforementioned single-cell transcriptomics data in *Kras*^*G12D*^-driven pancreatic tumors (Schlesinger et al. 2020) and found that endogenous *Kras* levels increased as the lesions progressed, peaking in the small OIS cluster and pancreatic ductal adenocarcinoma (PDAC) cells (Chan et al. 2024). Interestingly, such allelic imbalance with modest dosage increase is not limited to RAS: It is widespread with about half of all oncogenic driver mutations in a large advanced cancer cohort; thus, the concept of the OIS spectrum might also apply to other oncogenes (Bielski et al. 2018).

Altogether, it is possible that endogenous oncogenic RAS initially provides survival benefits to the cells, creating positive selective pressure, and gradually, cells with higher levels of oncogenic RAS become dominant. Once oncogenic RAS expression reaches a critical point, senescence may be induced, serving as a tumor-suppressive mechanism. At this point, some cells may progress toward malignancy due to other factors, such as mutations in tumor suppressor genes (Fig. 2B; Li et al. 2018; Chan and Narita 2019).

## OIS spectrum

To directly test this hypothesis, we recently applied single-cell profiling in the mouse liver OIS model (Chan et al. 2024). In this model, OIS is induced by ectopic expression of oncogenic NRAS^G12V^ through hydrodynamic tail vein (HDTV) injection (Kang et al. 2011). A unique feature of this model is that it recapitulates the interaction between senescent cells and immune cells: OIS hepatocytes trigger an immune response through the inflammatory SASP, and within 12–30 days after oncogenic *RAS* introduction, the senescent cells are eliminated by the immune cells (Kang et al. 2011; Pérez-Mancera et al. 2014; Yin et al. 2022). As a result, long-term liver cancer development is rarely observed. However, this model introduces considerable heterogeneity in the level of ectopic NRAS^G12V^; for example, at 6 days after the introduction of *NRAS*^*G12V*^ (when immune cell–mediated clearance has not yet occurred) (Chan et al. 2024). As in the pancreatic model described earlier (Schlesinger et al. 2020), single-cell transcriptomic data indicate that only a subset of hepatocytes exhibited characteristics consistent with classical OIS marked by *Cdk* inhibitors even in the ectopic NRAS^G12V^ model. Indeed, the OIS cluster corresponds to cells with the highest levels of ectopic *NRAS*. Therefore, this single-cell analysis provides a unique model to simultaneously investigate a broad range of ectopic oncogenic RAS levels, with OIS representing the end point of the spectrum in vivo.

**Figure 3.**
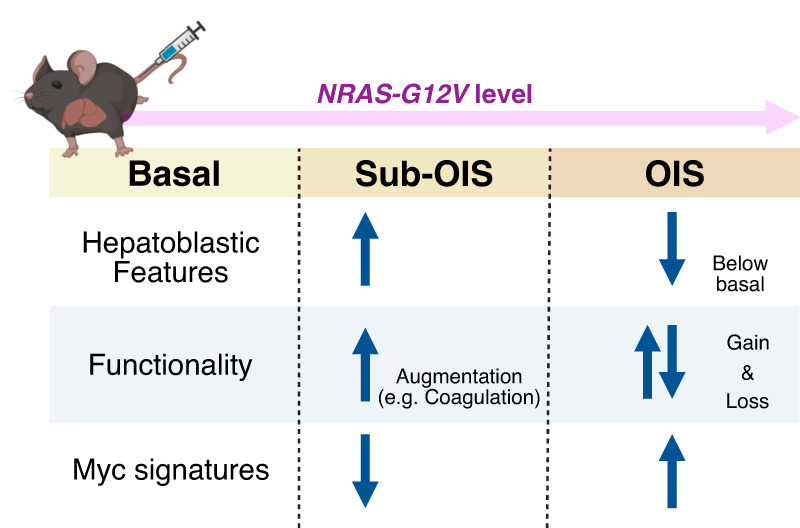
OIS spectrum in a mouse liver model. Hydrodynamic delivery of *NRAS*^*G12V*^ to hepatocytes using the strong CAGGS promoter results in hepatocytes with varying levels of ectopic NRAS^G12V^ expression. Previously, these cells were collectively referred to as senescent, but single-cell profiling defines “sub-OIS” clusters within the population. Some hepatocytic functions are enhanced in sub-OIS clusters (“functional augmentation”), including genes that are reduced even below basal levels in the OIS cluster (“loss of function”). See Figure 1 for an analogy to this process. (OIS) Oncogene-induced senescence.

Hepatocytes expressing relatively low levels of *NRAS* (“sub-OIS” clusters) showed hepatoblastic features, including progenitor and embryonic markers such as *Dlk1* and *Afp*, though *Afp* expression is more heterogeneous than *Dlk1*, which is strictly expressed by a small fraction of sub-OIS clusters. In addition, the hepatoblastic features of the OIS cluster were even lower than in the basal cluster, supporting our model that senescence epigenetically represents a more differentiated state (Fig. 3). Similarly, in the mice in which the *Kras*^*G12D*^ mutant is expressed at endogenous levels within the pancreas, sub-OIS cells also displayed upregulation of progenitor markers such as *Prom1*, *Pdx1*, and *Notch1*. However, such progenitor-like features were not obvious in the OIS cluster in this pancreatic model. Interestingly, our sub-OIS clusters were not just “hepatoblast-like” but also exhibited upregulation of genes encoding many hepatocytic secretory factors, including *Alb* (encoding albumin, also expressed in the fetal liver), *Apoa2* (apolipoprotein A-II), and *Fga* (fibrinogen α chain). This is reminiscent of the functional augmentation associated with the senescence transition phase that is characterized by NOTCH/TGFβ signaling during OIS in cultured HDFs (Hoare et al. 2016). Interestingly, some apolipoprotein-encoding genes, such as *Apoa2*, are rather downregulated in the OIS cluster, compared with the basal level, which may represent an aspect of the reduced functionality of the SASP. Considering the previously discussed similarities between cellular senescence and terminal differentiation, there seems to be a dynamic and nonlinear relationship between oncogenic RAS levels and cellular plasticity as well as functionality.

One notable factor that correlated with oncogenic RAS dosage in our study was Myc signaling. At the single-cell level, Myc target signatures exhibited a negative correlation with the *NRAS* level, except in OIS cells, which showed high levels of both *NRAS* and Myc signatures. This observation was somewhat unexpected given the well-established cooperative model of tumorigenesis involving these two oncogenes: RAS can induce senescence, which is resistant to cell death, whereas MYC blocks senescence but sensitizes cells to apoptosis (Casacuberta-Serra et al. 2024). Notably, this classic view is largely based on rodent cell models. Indeed, a recent study in HDFs suggests that these two oncogenes cooperatively induce senescence (Lopes-Paciencia et al. 2024). Consistently, when we systematically examined both public and our own data sets of in vitro senescence models, we found that MYC target genes are typically upregulated in OIS but downregulated in replicative and DNA damage-induced senescent cells. Additionally, overexpression of oncogenic HRAS in other human diploid cells (RPE1 cells) induced senescence-like features while maintaining proliferative capacity (a “slow-cycling” phenotype). Unlike OIS fibroblasts, these oncogenic RAS-induced slow-cycling cells showed downregulation of MYC targets, reinforcing our in vivo data that coactivation of MYC and RAS is a unique feature of OIS (Chan et al. 2024). Interestingly, Myc and Ras reciprocity was recently detected at a clonal level in a polyclonal intestine adenoma model in mice (Sadien et al. 2024). This suggests that an intricate relationship between RAS and MYC dosage—in either a cell-autonomous or a non-cell-autonomous fashion—may be integral to the OIS spectrum.

## Tumor initiation in the OIS spectrum

In the original mouse liver OIS model, the expression of oncogenic RAS was controlled by a strong promoter: CAGGS. When we titered down the expression using relatively weaker promoters (PGK and UBC), the phenotype was considerably different (Chan et al. 2024). In the short term, NRAS^G12V^-expressing hepatocytes largely persist in these settings. Strikingly, unlike the CAGGS-*NRAS*^*G12V*^ group, these low-dose oncogenic RAS mice—either the PGK-*NRAS*^*G12V*^ group or the UBC-*NRAS*^*G12V*^ group—develop liver cancer with high penetration (95% by ∼300 days after *NRAS*^*G12V*^ transduction). These results indicate that oncogenic RAS dosage is critical for senescence induction, immune surveillance, and tumor initiation.

**Figure 4.**
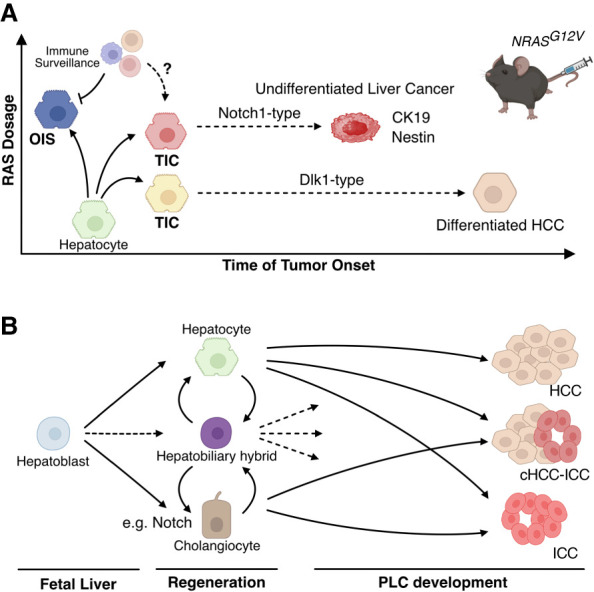
Two distinct tumor-initiating cell populations in the OIS spectrum in the liver. (*A*) OIS cells with high levels of NRAS^G12V^ are eliminated by immune cells. In contrast, tumor-initiating cell (TIC) candidates with low NRAS^G12V^ expression are characterized by *Dlk1* and other fetal liver markers, such as *Afp* and *Gpc3*, and are associated with late-onset, differentiated hepatocellular carcinoma (HCC). In contrast, TIC candidates with relatively high NRAS^G12V^ expression are associated with Notch and Tgfβ signaling as well as *Nes* (Nestin) and potentially give rise to aggressive, undifferentiated liver cancer. These TICs are typically surrounded by diverse immune cells. Although these tumors do not exhibit clear pathological features of intrahepatic cholangiocarcinoma (ICC), they show molecular features of ICC, including CK19. These two tumor types are highly distinct: Even when present within the same tumor, each occupies a separate area. Direct validation of these TIC candidates remains to be conducted, as indicated by dashed arrows. (*B*) A simplified view of cellular hierarchy in liver development and primary liver cancer (PLC) development. During fetal liver development, bipotential hepatoblasts differentiate into both hepatocytes and cholangiocytes, with the latter process promoted by, e.g., Notch signaling. Depending on the type of injury, hepatic progenitor cells (HPCs) or hepatobiliary hybrid cells can arise from either hepatocytes or cholangiocytes. Recently, resident hepatobiliary hybrid cells have been identified, and for simplicity, both types of bipotential cells are grouped together. Transdifferentiation between hepatocytes and cholangiocytes has been reported (not shown). Hepatocytes can give rise to HCC, ICC, and combined HCC–ICC (cHCC–ICC), whereas cholangiocytes contribute to ICC and cHCC–ICC. The cell of origin for each cancer type is highly context-dependent and remains somewhat ambiguous. HPCs have been proposed to contribute to all three cancer types, but their role remains debated, as indicated by dashed arrows.

To further investigate the relationship between OIS and sub-OIS, we performed single-cell analysis before (days 12 and 30 after oncogenic *NRAS*^*G12V*^ introduction) and after (day 218) tumor formation in the long-term observation of the UBC-*NRAS*^*G12V*^ mouse cohort. A pseudotime inference analysis integrating these physical time points revealed three main trajectories (Fig. 4A). One of these trajectories still exhibited relatively high *NRAS* levels and showed characteristics of OIS, including upregulation of *Cdkn2a*/*p16* and *Cdkn2b*/*p15*. This trajectory had no tumor components, reinforcing the tumor-suppressive role of OIS. A substantial part of the OIS cluster was observed at day 30 after *NRAS*^*G12V*^ injection—a time point when virtually all NRAS^G12V^-expressing cells are eliminated in the OIS-predominant CAGGS-*NRAS*^*G12V*^ setting; thus, those OIS cells in the UBC setting somehow escape immune surveillance.

The other trajectories were directed toward tumor cells but showed highly distinct gene expression profiles. The sub-OIS cells (d12 and d30) in these trajectories were associated with either (1) Notch and TGFβ signaling and *Nes* (encoding Nestin) expression or (2) *Dlk1*, *Gpc3*, and *Afp* expression. *Notch1* and *Dlk1*, for example, are largely exclusive of each other, possibly representing two distinct TIC candidates. Indeed, fully developed tumors in the UBC/PGK-*NRAS*^*G12V*^ cohorts shared some markers with either TIC type. Our pathological analyses identified liver tumors with varying levels of differentiation. One subset displayed undifferentiated, early-onset tumors that consistently expressed Notch1 and/or Nestin. The remaining tumor-burdened mice exhibited differentiated tumors, classified as hepatocellular carcinomas (HCCs), with longer latency and often positive for Dkl1. These data support the trajectory inference in the scRNA-seq analysis, though further experimental validation would be needed to confirm whether or not (a subset of) sub-OIS cells expressing individual progenitor markers are the direct precursors of individual tumor types (Fig. 4A).

Notably, whereas cells in the Notch1 TIC/tumor branch express ectopic *NRAS* at a level comparable with the OIS cluster, the Dlk1-type TIC/tumor branch shows a much lower *NRAS* expression. Immunohistological analysis also indicated that Notch1-positive tumors are strongly positive for NRAS. Consistently, our previous study demonstrated that a combination of a high level of ectopic NRAS^G12V^ and NOTCH intracellular domain (NICD), a constitutively active form of NOTCH, induces similar undifferentiated liver tumors, which are also positive for Nestin (Hoare et al. 2016; Chan et al. 2024). These suggest that RAS levels may influence not only OIS but also types of TICs, while it is not entirely clear how the Notch1 TICs bypass OIS or senescence surveillance.

## Primary liver cancer types and oncogenic RAS signaling

Hepatocellular carcinoma (HCC) and intrahepatic cholangiocarcinoma (ICC) are two major types of primary liver cancer (PLC), each exhibiting distinct morphological, molecular, and clinical characteristics, whereas combined HCC–ICC (cHCC–ICC) is a rare type of PLC that contains both HCC and ICC components. Common features of these types of PLC include that the tumor is typically preceded by chronic inflammation within the liver (Marquardt et al. 2015; Li et al. 2021; Gurzu et al. 2024; Zhang et al. 2024). As an example of the distinction between the PLC types, oncogenic *RAS* mutations are rare in HCC despite the general activation of RAS–MAPK signaling (Calvisi et al. 2006), whereas ICC frequently harbors oncogenic *KRAS* mutations, particularly at codon 12 (Zou et al. 2014).

There are three major epithelial cell types in the adult liver under homeostatic conditions: mature hepatocytes, biliary epithelial cells (BECs or cholangiocytes), and periportal bipotential hepatobiliary hybrid cells (Lotto et al. 2023). However, depending on the type of liver injury, hepatocytes and cholangiocytes also have a significant regenerative capacity and plasticity with the ability to not only give rise to progenitor-like cells but also transdifferentiate into biliary-like cells and hepatocytes, respectively (Deng et al. 2018; Schaub et al. 2018; Lotto et al. 2023).

Various genetically engineered and fate-tracing mouse models have been used to determine the potential cell of origin of specific tumor types. Although it is recognized that hepatic progenitor cells contribute to the development of PLC, some fate-tracing animal studies suggest that HCC rarely originates from resident hepatobiliary hybrids or hepatic progenitor cells at least in some HCC models in mice (Font-Burgada et al. 2015; Mu et al. 2015; Sia et al. 2017). This is in part due to a lack of clear consensus on the identity of the resident and induced hepatobiliary hybrids and the diversity of their plasticity (Lotto et al. 2023). However, strong evidence suggests that hepatocytes can give rise to both HCC and ICC, whereas ICC can also derive from the cholangiocytes themselves (Fan et al. 2012; Sekiya and Suzuki 2012), particularly under conditions of chronic biliary damage and inflammation (Fig. 4B; Li et al. 2021). Early tumor-initiating events, however, are still elusive.

Our UBC/PGK-*NRAS*^*G12V*^ model uses HDTV delivery of ectopic genes that are targeted primarily to hepatocytes, reinforcing the plasticity of hepatocytes, which can be a source of diverse PLCs. Although the Notch1-positive tumors in the UBC/PGK-*NRAS*^*G12V*^ models lack the typical ICC morphology, they are pathologically undifferentiated, with molecular features that overlap with ICC, including positivity for a marker of the hepatobiliary tract, cytokeratin 19 (CK19). Indeed, NOTCH signaling itself has been linked to ICC, and ectopic expression of constitutively active NOTCH (NICD) in the fetal liver can induce ICC development in mice (Zender et al. 2013). In addition, Nestin has been implicated in aggressive, poorly differentiated liver cancer in humans and mice (Tschaharganeh et al. 2014) and found elevated particularly in cHCC–ICC (Xue et al. 2019). Thus, we speculate that although Dlk1-type TICs develop into differentiated HCCs, Notch1-type TICs may give rise to aggressive undifferentiated ICC-like tumors. This pattern intriguingly parallels fetal liver development, where Dlk1 marks bipotential hepatoblasts (which can be differentiated into both hepatocytes and cholangiocytes), and Notch/Tgfβ signaling promotes cholangiocyte differentiation (Notch1-type TICs also show elevated Tgfβ signaling) (Miyajima et al. 2014). Notch signaling is also implicated in the biliary specification of the hepatic progenitor cells during chronic liver injury (Fig. 4B; Boulter et al. 2012). Our data that Notch1-associated undifferentiated tumors tend to express higher levels of ectopic NRAS^G12V^ than the other type may also be consistent with the high prevalence of oncogenic *KRAS* mutations in human ICC but not in HCC (Chan et al. 2024).

## Early immune response in low-dose oncogenic RAS context in livers

One notable early distinction between high-dosage (CAGGS promoter-driven) and reduced-dosage (UBC/PGK promoter-driven) oncogenic RAS models in the liver is the extent of immune surveillance. The former (an OIS model) shows effective immune clearance, but the precise mechanisms underlying the immune resistance of NRAS^G12V^-expressing hepatocytes in the latter remain to be determined. In our scRNA-seq from the CAGGS-*NRAS*^*G12V*^ mice, several critical effectors, such as *Ccl2* and *Ptgs2*, for OIS surveillance are induced in the OIS culture, but such induction is weaker or absent in sub-OIS hepatocytes (Chan et al. 2024). For example, Ccl2, a SASP component, is essential for the recruitment of immature monocytes and thus senescence surveillance (Eggert et al. 2016). We recently showed that Cox2 (encoded by *Ptgs2*), a key enzyme for producing prostaglandins, is upregulated during senescence and activates inflammatory SASP partly through autocrine feedback involving prostaglandin E2 (PGE2) (Gonçalves et al. 2021). Consistent with the weaker induction of *Ccl2* and *Ptgs2* in sub-OIS hepatocytes, unlike the OIS (CAGGS-*NRAS*^*G12V*^) model, the global immune phenotyping using flow cytometry exhibited minimum T-cell response in PGK/UBC-*NRAS*^*G12V*^ mice (Chan et al. 2024).

However, the reduction or lack of immune surveillance in response to lower levels of oncogenic NRAS in the liver is not simply due to the weaker T-cell response. In the CAGGS-*NRAS*^*G12V*^ context, immune clusters, where NRAS^G12V^-expressing hepatocytes are surrounded by T cells and macrophages, can often be observed before elimination. In the UBC-*NRAS*^*G12V*^ and, more prominently, PGK-*NRAS*^*G12V*^ models, similar but more pronounced immune cell clusters are observed even after day 12, suggesting an overall reduction in immune response with focal enrichment of immune cells. Interestingly, these persistent immune clusters primarily involved Notch1-positive hepatocytes, whereas Dlk1-positive hepatocytes were largely excluded. Thus, these two TIC candidates (NOTCH type and Dlk1 type) also lead to distinct immune microenvironments.

The biological significance of persistent immune clusters surrounding Notch1-positive sub-OIS hepatocytes is unclear. These cells typically express high levels of NRAS^G12V^ but somehow escape OIS and immune surveillance. In OIS HDF models in culture, activation of endogenous NOTCH signaling represents early and perhaps incomplete senescence (Fig. 1; Hoare et al. 2016; Olan et al. 2024). Indeed, NOTCH signaling negatively controls inflammatory SASP and SAHF formation during HDF OIS (Hoare et al. 2016; Parry et al. 2018). In addition, we previously showed that inhibiting NOTCH-responsive gene expression by ectopic expression of a dominant negative form of coactivator mastermind1 (dnMAML1) together with CAGGS-*NRAS*^*G12V*^ promotes OIS surveillance (Hoare et al. 2016). We speculate that Notch and possibly other effectors, such as Tgfβ signaling, may buffer the senescence phenotype by promoting plasticity and altering the secretory program. In addition, such persistent immune clusters may provide a unique niche, thus shaping the TICs in a non-cell-autonomous manner. Strikingly, the majority of examined human cirrhotic livers, which represent a high-risk lesion for PLC, contained NOTCH1- and/or DLK1-positive hepatocytes, and NOTCH1-positive hepatocytes in particular were readily identified due to their involvement in similar immune clusters seen in mice.

## Conclusion

In parallel to the current descriptive, multimarker approach for defining senescence, we propose an overarching definition of senescence as a stable shift of cell fate. Senescence is not simply a cellular degenerative process (loss of function). It also represents a new functional identity (gain of function). However, similar to terminal differentiation, this process is not binary but rather a continuum. More importantly, it is not a linear process, highlighting the importance of understanding “subsenescence” states. Although this is largely based on the OIS models, the same principle may apply to other types of senescence.

In the OIS context, we have focused on the relationship between oncogenic dosage and early phenotypes. However, other factors are undoubtedly involved. For example, p53, a key transcription factor in senescence, has a repressive role in Nestin expression in liver progenitor cells, and loss of p53 (thus, Nestin upregulation) together with either WNT or NOTCH signaling promotes HCC or ICC development, respectively (Tschaharganeh et al. 2014). MYC levels have also been shown to affect the lineage commitment in PLC, where high levels of Myc drive HCC formation by activating Forkhead family transcription factors FOXA1 and FOXA2, while low MYC levels allow ETS1 to promote ICC development (D'Artista et al. 2023). Notably, we showed a negative correlation between *NRAS* and Myc signatures in sub-OIS hepatocytes, where Notch1-expressing cells showed low levels of Myc signatures (Chan et al. 2024). Cell fate determination is also affected through a complex interplay with the microenvironment: SASP factors derived from senescent stromal cells modulate HCC development. Yoshimoto et al. (2013) demonstrated that obesity-associated HCC is promoted by the SASP of senescent hepatic stellate cells (HSCs), a major source of senescent cells in chronic liver damage in mice (Krizhanovsky et al. 2008). Notably, the protumorigenic effects of stromal senescence appear to be context-dependent, as Lujambio et al. (2013) have shown a tumor-suppressive role of senescent HSCs using a mouse model where they combined a carcinogen and a fibrogenic agent. In addition to the different background pathogens, it was suggested that p53 status in both epithelial and stromal cells might modulate the relationship between tumor-initiating cells and stromal components (Ohtani et al. 2014). Moreover, Seehawer et al. (2018) have shown that the same oncogenic mutations in hepatocytes drive either HCC or ICC with an apoptotic or a necroptosis-associated microenvironment, respectively.

The OIS spectrum idea may have therapeutic implications as an early intervention. For example, manipulating the activity of the RAS–MAPK pathway or NOTCH signaling may impact the TIC trajectories. We have shown that reducing MAPK activity by sorafenib, a multikinase inhibitor that targets RAF and several upstream receptor tyrosine kinases, in our UBC-*NRAS*^*G12V*^ mice had no effect on immune surveillance but inhibited the formation of granuloma-like immune clusters, while its long-term impact on tumor development remains to be determined (Chan et al. 2024). Selective killing of senescent cells (or senolytics) has gained tremendous attention. The heterogeneity of senescence may also impact the senolytics strategy, which currently targets senescence as a bulk population. It is not clear whether or not those senolytics drugs can eliminate sub-OIS cells or TICs. It is also possible that the selective elimination of the OIS cluster may alter the immune landscape: Given the lack of efficient immune surveillance in our sub-OIS-predominant (UBC-*NRAS*^*G12V*^ or PGK-*NRAS*^*G12V*^) model, despite a substantial OIS induction, there might be a “threshold” for OIS cell abundance required to trigger effective “bystander” immune surveillance for TICs. It is also possible that SASP factors from persistent OIS cells promote tumorigenesis. Thus, OIS can act as both an autonomous tumor-suppressive and a nonautonomous tumorigenic mechanism. These could partly explain why endogenous oncogenic Kras models, whether in the pancreas or the lung, do not exhibit robust senescence surveillance and eventually develop tumors. We hope that a deeper understanding of senescence diversity will pave the way for new cancer prevention strategies.
